# Administering the maternal bovine appeasing substance improves fertility in lactating dairy cows

**DOI:** 10.3168/jdsc.2025-0876

**Published:** 2025-12-04

**Authors:** Martin Zinicola, Reinaldo F. Cooke, Francisco Larghi

**Affiliations:** 1 Cowix, San Francisco, Córdoba 2400, Argentina; 2 Department of Animal Sciences, Texas A&M University, College Station, TX 77843

## Abstract

This experiment evaluated the effects of administering the maternal bovine appeasing substance (mBAS) at the time of artificial insemination (AI) on pregnancy per AI (P/AI) of Holstein cows under commercial dairy conditions. A total of 375 lactating Holstein dairy cows (178 primiparous and 197 multiparous cows) from 2 commercial dairy farms were enrolled. All cows were assigned to an ovulation synchronization + AI protocol when they reached 65 DIM. The protocol included 2 mg of estradiol benzoate + intravaginal progesterone-releasing device (DIB) on d −10, followed by 25 mg of dinoprost tromethamine on d −3, followed by 25 mg of dinoprost tromethamine + 1 mg of estradiol cypionate + 400 IU of equine chorionic gonadotropin + DIB removal and tail paint on d −2. On d 0 (24 h after tail paint application), cows with paint removed were classified as in estrus and inseminated. Cows with intact tail paint were considered not to be in estrus, administered 100 µg of GnRH, and inseminated 8 h later. At the time of AI, cows within farm and parity were randomly assigned to receive mBAS (Ferappease, FERA Diagnostics and Biologicals, College Station, TX; n = 197) or no treatment (CON, n = 178). The mBAS (10 mL) was applied topically to the nuchal skin area (5 mL) and above the muzzle (5 mL). Pregnancy status to AI was verified by detecting a viable embryo with transrectal ultrasonography 32 d after AI. Cows assigned to mBAS or CON had similar DIM at the time of AI, milk yield, and incidence of estrus during the synchronization protocol. Administering mBAS increased P/AI by 12.5 percentage points compared with CON cows (60.2% vs. 47.7%). Based on treatment differences in P/AI and an economic value of $200/pregnancy, mBAS administration yielded a return-on-investment of 833%. This calculation assumed mBAS cost of $3.00/cow, a herd of 1,000 cows in which mBAS yielded 125 more pregnancies, resulting in an economic benefit of $25,000 ($25,000/$3,000 = 8.33). The results of this experiment are novel and provide evidence that mBAS is effective in enhancing the reproductive performance of lactating dairy cows when applied during critical stress-sensitive periods such as AI.

## Highlights

• Stress near the time of AI decreases fertility in dairy cows.
• mBAS attenuates stress-related responses in cattle.
• Administering this substance at AI increased pregnancy by 26% in dairy cows.
• This increase in pregnancy may result in a return-on-investment of 833%.

## Graphical Abstract

**Figure:**
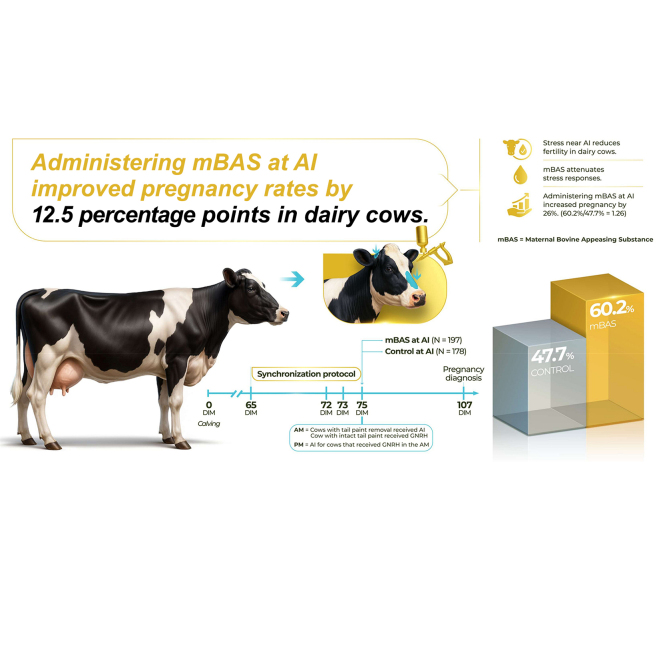
**Summary:** The maternal bovine appeasing substance (mBAS) replicates the composition of the original bovine appeasing pheromone and has been shown to attenuate stress-related behavioral and physiological responses in cattle. Stress near the time of artificial insemination (AI) reduces conception rates, underscoring the need for interventions targeting this critical period. In this experiment, 375 lactating Holstein cows were assigned to an ovulation synchronization + AI protocol when they reached 65 days in milk (DIM). Cows received mBAS (197 cows) or not (control; 178 cows) at the time of AI. The mBAS was applied topically to the nuchal skin area (5 mL) and above the muzzle (5 mL) of each cow. Pregnancy per AI increased by 12.5 percentage points in mBAS treated cows (60.2% compared with 47.7% for mBAS and no treatment-cows, respectively). Results from this experiment are novel and indicate that mBAS is an effective tool to enhance the reproductive performance of dairy cows when applied during critical stress-sensitive periods such as AI. GNRH = gonadotropin-releasing hormone.

Reproductive efficiency in lactating dairy cows is critical to the sustainability and profitability of dairy operations, as it directly influences milk production, the generation of replacement heifers, and rates of involuntary culling (Walsh et al., 2011). Despite decades of research that have advanced the understanding of bovine fertility and led to improvements in reproductive management, modern dairy farms continue to encounter persistent challenges (Lucy and Pohler, 2025). These include market volatility, labor shortages, narrow profit margins, and the escalating global demand for animal protein, all of which necessitate continual optimization of herd performance and management systems (Wemette et al., 2021; Ufer and Ortega, 2024).

Among the numerous factors affecting reproductive success, stress has gained recognition as a significant contributor to subfertility. Stressful conditions—including handling, social instability, climatic stress, and metabolic load—can disrupt endocrine homeostasis and impair the hypothalamic-pituitary-gonadal axis, resulting in diminished ovulation, fertilization, or embryo survival (Dobson and Smith, 2000; Dobson et al., 2001). Notably, acute stress events around the time of artificial insemination (**AI**) have been associated with reduced conception rates, underscoring the need for interventions targeting this critical period (von Borell et al., 2007; Cooke et al., 2017).

Recent attention has turned toward nonantibiotic, nonhormonal strategies to mitigate stress and enhance fertility in cattle. One such approach involves the maternal bovine appeasing substance (**mBAS**), which includes a mixture of fatty acids that replicate the composition of the original bovine appeasing pheromone (Cappellozza and Cooke, 2022). The mBAS has been shown to attenuate stress-related behavioral and physiological responses in cattle, including beef and dairy calves (Bringhenti et al., 2023; Pickett et al., 2024) as well as mature feedlot steers (Cooke et al., 2025; Mackey et al., 2025). By reducing stress at key management points, particularly during AI, mBAS may support improved reproductive performance and overall productivity in dairy systems. Based on this rationale, we hypothesized that administration of mBAS at the time of AI will increase pregnancy per AI (**P/AI**) in dairy cows. Therefore, the objective of this experiment was to evaluate the impact of mBAS administration at the time of first-service AI on pregnancy rates of lactating Holstein cows under commercial dairy conditions.

This experiment was conducted in 2 commercial dairy farms located in central Argentina (Marull, Córdoba). The animals used in these experiments were cared for in accordance with acceptable practices as outlined in the *Guide for the Care and Use of Agricultural Animals in Research and Teaching* (FASS, 2020). All cows were managed in drylot according to the existing nutritional, reproductive, and sanitary procedures of each operation. Cows received a TMR for ad libitum consumption, formulated to meet or exceed their nutritional requirements according to parity, DIM, and milk production (NASEM, 2021).

A total of 375 lactating Holstein dairy cows (178 primiparous and 197 multiparous cows) were enrolled into the experiment, as described in Figure 1. This sample size provided a statistical power of 0.63, based on a 10 percentage point difference in P/AI between treatments (i.e., 50% vs. 60%), using the G*power 3 software (z-test; Faul et al., 2007). This experimental design provided moderate statistical power to detect biologically meaningful differences in P/AI under field conditions, which is below the conventional 0.80 benchmark, and represents a preliminary assessment of the potential fertility benefits of mBAS in dairy cows. All cows were assigned to an ovulation synchronization + AI protocol when they reached 65 DIM. More specifically, the protocol (d −10 to 0) included 2 mg of estradiol benzoate (Gonadiol, Zoetis, Buenos Aires, Argentina) + bovine intravaginal progesterone-releasing device (**DIB**; Zoetis) on d −10, followed by 25 mg of dinoprost tromethamine (Lutalyse, Zoetis) on d −3, followed by 25 mg of dinoprost tromethamine (Lutalyse, Zoetis) + 1 mg of estradiol cypionate (Cipiosyn, Zoetis) + 400 IU of equine chorionic gonadotropin (Novormon, Zoetis) + DIB removal and application of tail paint for estrus detection (BoviMarker, Santa Fé, Argentina) on d −2. On d 0 (24 h after tail paint application), cows with paint removed were classified as in estrus and inseminated. At the same time, cows with intact tail paint were considered not to be in estrus and were administered 100 µg of GnRH (Ovusyn**;** Zoetis), and then inseminated 8 h later (Figure 1). Cows were inseminated using conventional semen from Holstein sires and by technicians that were randomly allocated to cows within each farm.

**Figure 1:**
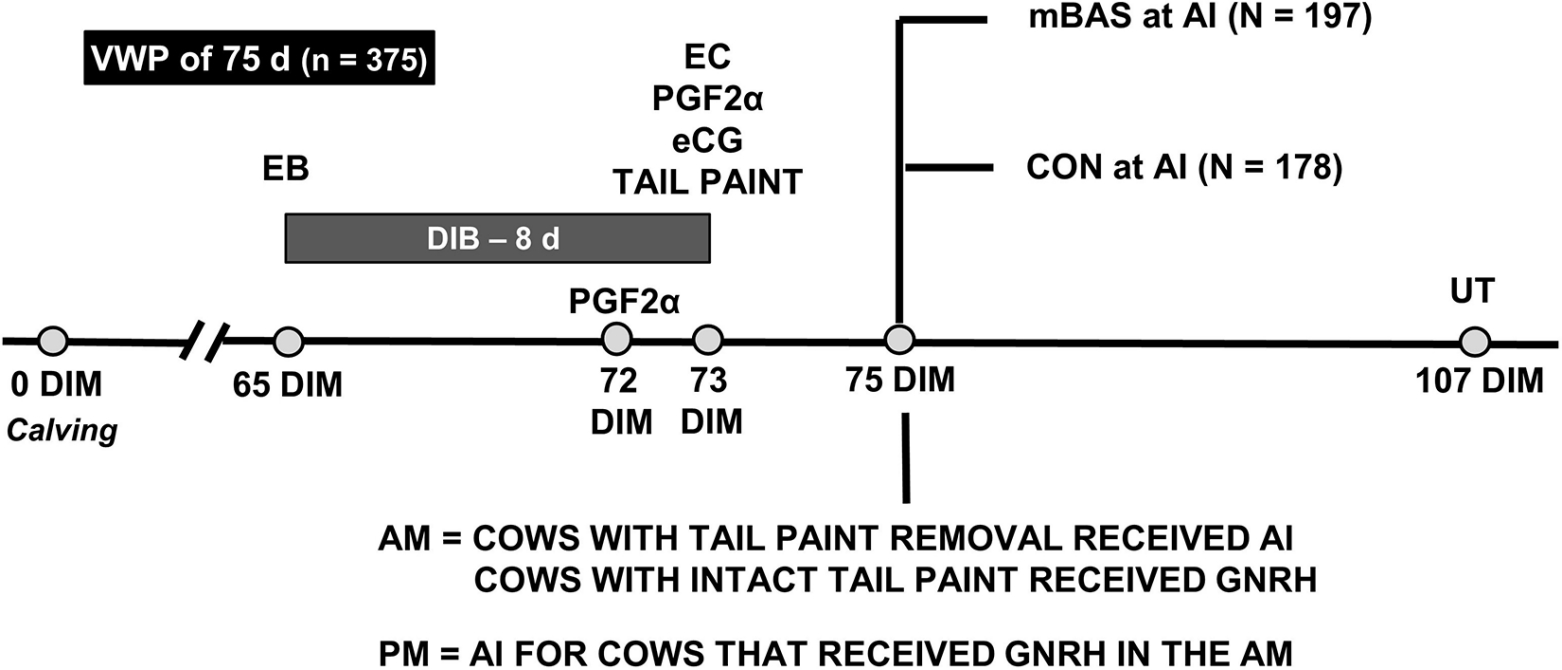
Experimental design: 375 lactating Holstein cows from 2 commercial farms were assigned to this experiment. At the time of AI, cows within farm and parity were randomly assigned to receive mBAS (Ferappease, FERA Diagnostics and Biologicals, College Station, TX; n = 197) or no treatment (CON, n = 178). EB = estradiol benzoate; EC = estradiol cypionate; DIB = intravaginal progesterone-releasing device; eCG = equine chorionic gonadotropin; UT = ultrasonography; VWP = voluntary waiting period.

At the time of AI, cows within farm and parity were randomly assigned to receive mBAS (Ferappease, FERA Diagnostics and Biologicals, College Station, TX; n = 197) or no treatment (**CON**, n = 178). The active ingredient of mBAS is based on a proprietary mixture of fatty acids including palmitic, oleic, and linoleic acids, and added at 10% of the excipient (Pickett et al., 2024). The mBAS (10 mL) was applied topically to the nuchal skin area (5 mL) where intradermal sebaceous glands absorb the product, release back to the skin along with the natural sebum for 15 d, and can readily reach the nostrils (Cappellozza and Cooke, 2022). Another 5 mL is applied above the muzzle (5 mL) for prompt delivery of mBAS near the nostrils. Both areas receive mBAS using the applicator provided by the manufacturer (FERA Diagnostics and Biologicals). Cows were alternately assigned to receive mBAS and CON as they entered the palpation rail (capacity for 20 cows at a time), whereas mBAS cows received treatment and AI with a 5 m distance from CON cows to minimize cross-contamination. Cows assigned to mBAS and CON within farm were housed in the same pen before treatment application, and returned to same pen immediately after AI.

Pregnancy status to AI was verified by detecting a viable embryo with heartbeat using transrectal ultrasonography 32 d after AI. Milk yield during the first and second month of lactation was obtained from farm records, and based on monthly DHIA test-day milk yield of each cow. Data were analyzed using cow as the experimental unit, and Satterthwaite approximation to determine the denominator df for the tests of fixed effects. Quantitative data were analyzed with the MIXED procedure of SAS (SAS Institute Inc., Cary, NC), and binary data were analyzed with the GLIMMIX procedure of SAS with a binomial distribution and logit link function. The model statement used for DIM, milk yield, and incidence of estrus contained the fixed effects of treatment, parity, and the resultant interaction. The model statement used for P/AI contained the fixed effects of treatment, parity, incidence of estrus, and all resultant interactions. The random statement for all models contained the effects of cow (treatment × parity × farm), sire (farm), technician (farm), and farm as random variables. Significance was set at *P* ≤ 0.05 and tendencies were determined if *P* > 0.05 and ≤0.10. Results are reported as least squares means according to the effect of treatment if no interactions were significant, or according to the highest-order interaction detected.

No differences between mBAS and CON cows were detected (*P* ≥ 0.22) for DIM at the time of AI, milk yield, and incidence of estrus during the synchronization protocol (Table 1). No treatment × parity interactions were detected (*P* ≥ 0.42) for any of these variables. Cows assigned to mBAS had greater (*P* = 0.02) P/AI compared with CON cows (Figure 2). No treatment × parity, treatment × estrus incidence, nor treatment × parity × estrus incidence interactions were detected (*P* ≥ 0.22) for P/AI. These results are novel and demonstrate mBAS at the time of first postpartum AI improved reproductive performance of lactating Holstein cows under commercial conditions. Specifically, mBAS administration increased P/AI by 12.5 percentage points, which corresponds to a 26% increase (60.2%/47.7% = 1.26), compared with CON cows. These findings support our hypothesis that reducing stress-related behavioral and physiological responses near the time of AI improved P/AI in dairy cows.

**Table 1:** General characteristics of lactating Holstein cows assigned to receive the maternal bovine appeasing substance (mBAS) or not (CON) at the time of AI^1^

Item	CON	mBAS	SEM	*P-*value
Cows, n	178	197	—	—
Primiparous	91	99	—	—
Multiparous	87	98	—	—
DIM	76.2	76.6	0.7	0.74
Milk yield, kg/d				
First month of lactation	42.7	42.9	0.6	0.82
Second month of lactation	44.1	44.9	0.6	0.29
Average	43.4	43.9	0.5	0.49
Incidence of estrus, %	65.2 (116/178)	71.0 (140/197)	3.5	0.22

1 Cows were assigned to ovulation synchronization + AI protocol when they reached 65 DIM. The protocol (d −10 to 0) included 2 mg of estradiol benzoate + bovine intravaginal progesterone-releasing device (DIB; Zoetis) on d −10, followed by 25 mg of dinoprost tromethamine on d −3, followed by 25 mg of dinoprost tromethamine + 1 mg of estradiol cypionate + 400 IU of equine chorionic gonadotropin + DIB removal and application of tail paint for estrus detection on d −2. On d 0 (24 h after paint application), cows with paint removed were classified as in estrus and inseminated. At the same time, cows with intact tail paint were considered not to be in estrus and were administered 100 µg of GnRH, and then inseminated 8 h later. At the time of AI, cows within farm and parity were randomly assigned to receive 10 mL of mBAS (Ferappease, FERA Diagnostics and Biologicals, College Station, TX; n = 197) or no treatment (CON, n = 178).

**Figure 2:**
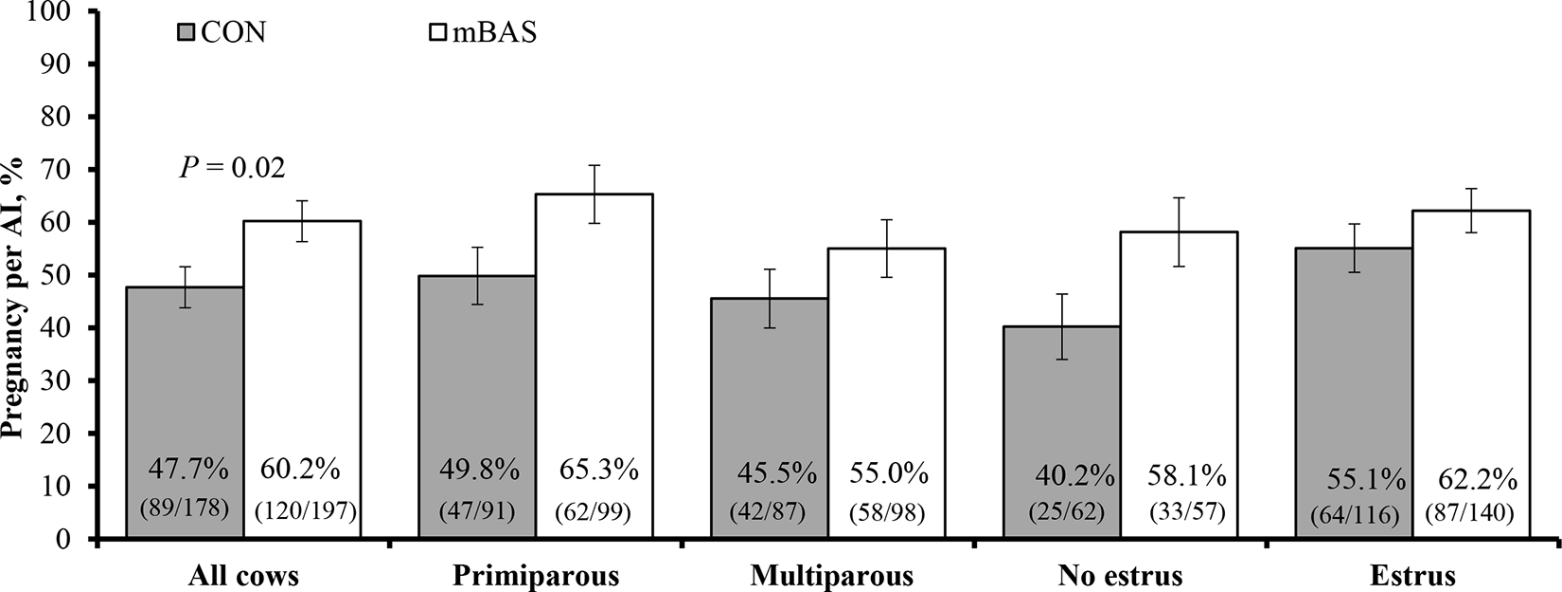
Pregnancy per AI in lactating Holstein cows assigned to receive 10 mL of the maternal bovine appeasing substance (mBAS; Ferappease, FERA Diagnostics and Biologicals, College Station, TX; n = 197) or not (CON; n = 178) at the time of AI. Cows were assigned to ovulation synchronization + AI protocol that included 2 mg of estradiol benzoate + bovine intravaginal progesterone-releasing device (DIB) on d −10, followed by 25 mg of dinoprost tromethamine on d −3, followed by 25 mg of dinoprost tromethamine + 1 mg of estradiol cypionate + 400 IU of equine chorionic gonadotropin + DIB removal and tail paint on d −2. On d 0 (24 h after paint application), cows with paint removed were classified as in estrus and inseminated. Cows with intact tail paint were considered not to be in estrus and administered 100 µg of GnRH, and then inseminated 8 h later. A treatment effect was detected (*P* = 0.02), whereas the treatment × parity, treatment × estrus incidence, and treatment × parity × estrus incidence interactions were not significant (*P* ≥ 0.22). Error bars represent SEM.

Acute stress is known to impair the hypothalamic-pituitary-gonadal axis, interfere with luteinizing hormone secretion, and compromise ovulation and early embryo development (Dobson and Smith, 2000; Dobson et al., 2001). Behavioral or physiological stress at the time of AI reduces conception rates in cattle (von Borell et al., 2007; Cooke et al., 2017), and that even minor handling procedures can alter cortisol levels and reproductive hormone dynamics (Collier et al., 2017). Therefore, practical tools to mitigate the negative impacts of stress during AI hold considerable promise for improving fertility. The mBAS has previously been shown to reduce behavioral and physiological stress responses in cattle, including temperament score and cortisol concentration in plasma and hair from tail switch (Cappellozza and Cooke, 2022; Pickett et al., 2024). The present results expand on those findings by demonstrating a functional benefit of mBAS on reproductive performance of dairy cows. The improvement in P/AI aligns with the proposed mechanism of action, where reducing acute stress at a critical physiological window enhances the likelihood of conception (Dobson et al., 2001; Cooke et al., 2017).

The increase in P/AI by mBAS administration was observed across parities and incidence of estrus based on the lack of interactions among these variables with treatment effect. These outcomes provide evidence that the efficacy of mBAS is robust amid varying physiological conditions, which corroborates the value of mBAS in commercial dairy settings where stressors around AI are ubiquitous and often unavoidable (Bruckmaier et al., 1993; Rushen, 2017). Based on the economic value of $200/pregnancy proposed by Cabrera (2012) and treatment effects on P/AI noted herein, the return-on-investment by mBAS administration was estimated at 833%. This return-on-investment assumed a herd of 1,000 cows, in which mBAS administration would yield 125 more pregnancies (60.2% vs. 47.7% P/AI of mBAS and CON cows, respectively), resulting in an economic benefit of $25,000. The costs associated with mBAS administration were set at $3,000/herd ($3.00/cow for product cost; Pickett et al., 2024), resulting in the 833% return-on-investment ($25,000 economic increase divided by $3,000 of additional costs). Costs with additional labor for mBAS administration were not included in this calculation because these should be minimal, if any, as cows were already being handled for AI.

The mBAS and CON cows in this experiment were housed in the same pen within each farm before and after treatment administration as previously described. This approach is common in research evaluation of health and reproductive technologies in dairy cattle, such as vaccines (Pereira et al., 2013; Meira et al., 2020) and estrus synchronization protocols (Giordano et al., 2012; Wiltbank et al., 2015), exposing cows from different treatments to the exact same management and environmental conditions. It can be conjectured that CON cows were also exposed, at least to some extent, to mBAS by sharing the same pen with mBAS-treated cows. Nonetheless, mBAS increased P/AI despite this potential physical limitation; perhaps such improvements would be even greater if CON and mBAS cows were housed in different pens. Based on this rationale and the novelty of the results reported herein, research is warranted to further explore and validate the benefits of mBAS administration to reproductive efficiency of lactating dairy cows.

Collectively, mBAS administration at the time of AI significantly improved fertility outcomes in lactating Holstein cows, suggesting that mBAS may be an effective tool to enhance reproductive performance when applied during critical stress-sensitive periods such as AI. The beneficial effects of mBAS were noted across lactation groups and incidence of estrus, indicating its broad applicability under commercial dairy conditions. The overall improvement in fertility was estimated to yield a ROI of 833%, meaning that each $1.00 invested in mBAS resulted in $8.30 of economic benefits. Modern dairy systems are marked by labor constraints, economic pressures, and a demand for nonantibiotic, welfare-friendly management tools (Wemette et al., 2021; Ufer and Ortega, 2024). Therefore, the adoption of practical interventions such as mBAS offers a novel and effective strategy to support welfare, reproductive performance, overall efficiency, and profitability in dairy herds.
